# Xuebijing injection protects against sepsis-induced myocardial injury by regulating apoptosis and autophagy via mediation of PI3K/AKT/mTOR signaling pathway in rats

**DOI:** 10.18632/aging.204740

**Published:** 2023-05-22

**Authors:** Cheng-Fei Bi, jia Liu, Shao-Wen Hao, Zhi-Xia Xu, Xiao Ma, Xiang-Fei Kang, Li-Shan Yang, Jun-Fei Zhang

**Affiliations:** 1 Department of Emergency Medical, General Hospital of Ningxia Medical University, Yinchuan 750000, Ningxia, China; 2 Medical Experimental Center, General Hospital of Ningxia Medical University, Yinchuan 750000, Ningxia, China; 3 School of Clinical Medicine, Ningxia Medical University, Yinchuan 750000, Ningxia, China

**Keywords:** sepsis induced myocardial injury, Xuebijing injection, apoptosis, autophagy, PI3K/AKT/mTOR

## Abstract

Objective: Apoptosis and autophagy are significant factors of sepsis induced myocardial injury (SIMI). XBJ improves SIMI by PI3K/AKT/mTOR pathway. Present study is devised to explore the protective mechanism of XBJ in continuous treatment of SIMI caused by CLP.

Methods: Rat survival was first recorded within 7 days. Rats were randomly assigned to three groups: Sham group, CLP group, and XBJ group. The animals in each group were divided into 12 h group, 1 d, 2 d, 3 d and 5 d according to the administration time of 12 hours, 1 day, 2 days, 3 days or 5 days, respectively. Echocardiography, myocardial injury markers and H&E staining were used to detect cardiac function and injury. IL-1β, IL-6 and TNF-α in serum were measured using ELISA kits. Cardiomyocyte apoptosis was assayed by TUNEL staining. Apoptosis and autophagy related proteins regulated by the PI3K/AKT/mTOR signaling pathway were tested using western blot.

Results: XBJ increased the survival rate in CLP-induced septic Rat. First of all, the results of echocardiography, H&E staining and myocardial injury markers (cTnI, CK, and LDH levels) showed that XBJ could effectively improve the myocardial injury caused by CLP with the increase of treatment time. Moreover, XBJ significantly decreased the levels of serum inflammatory cytokines IL-1β, IL-6 and TNF-α in SIMI rats. Meanwhile, XBJ downregulated the expression of apoptosis-related proteins Bax, Cleaved-Caspase 3, Cleaved-Caspase 9, Cytochrome C and Cleaved-PARP, while upregulated the protein levels of Bcl-2 in SIMI rats. And, XBJ upregulated the expression of autophagy related protein Beclin-1 and LC3-II/LC3-I ratio in SIMI rats, whereas downregulated the expression of P62. Finally, XBJ administration downregulated the phosphorylation levels of proteins PI3K, AKT and mTOR in SIMI rats.

Conclusions: Our results showed that XBJ has a good protective effect on SIMI after continuous treatment, and it was speculated that it might be through inhibiting apoptosis and promoting autophagy via, at least partially, activating PI3K/AKT/mTOR pathway in the early stage of sepsis, as well as promoting apoptosis and inhibiting autophagy via suppressing PI3K/AKT/mTOR pathway in the late stage of sepsis.

## INTRODUCTION

Sepsis, a potentially fatal host reaction to infection that causes organ failure, is one of the primary diseases that poses a substantial threat to the health of people worldwide [1]. According to an epidemiological assessment conducted in 2020, sepsis has a global incidence and mortality rate of approximately 6.775% and 1.481%, respectively [2]. A significant sign of disease progression in hospitalized septic patients is sepsis-induced multiorgan dysfunction, which has a pooled hospital incidence of 9.3% [3] and an in-hospital mortality greater than 10% [1]. It has drawn more attention in recent years in the field of Intensive Care Unit (ICU) management among the numerous secondary organ dysfunctions associated with sepsis, and myocardial injury is one of the most frequent complications and a significant cause of death in septic patients [4]. Additionally, myocardial injury, which has incidence of between 10 and 70% [4] and the fatality rate of 70–90% [1], predominates in the pathophysiology of sepsis. Although there have been significant improvements in organ function support, stabilization of microcirculation, and anti-infective therapy in recent years [5, 6], there are still no satisfactory medications or therapies for sepsis. As a result, finding new cures to treat SIMI is challenging but essential for managing sepsis patients.

**Figure 1:**
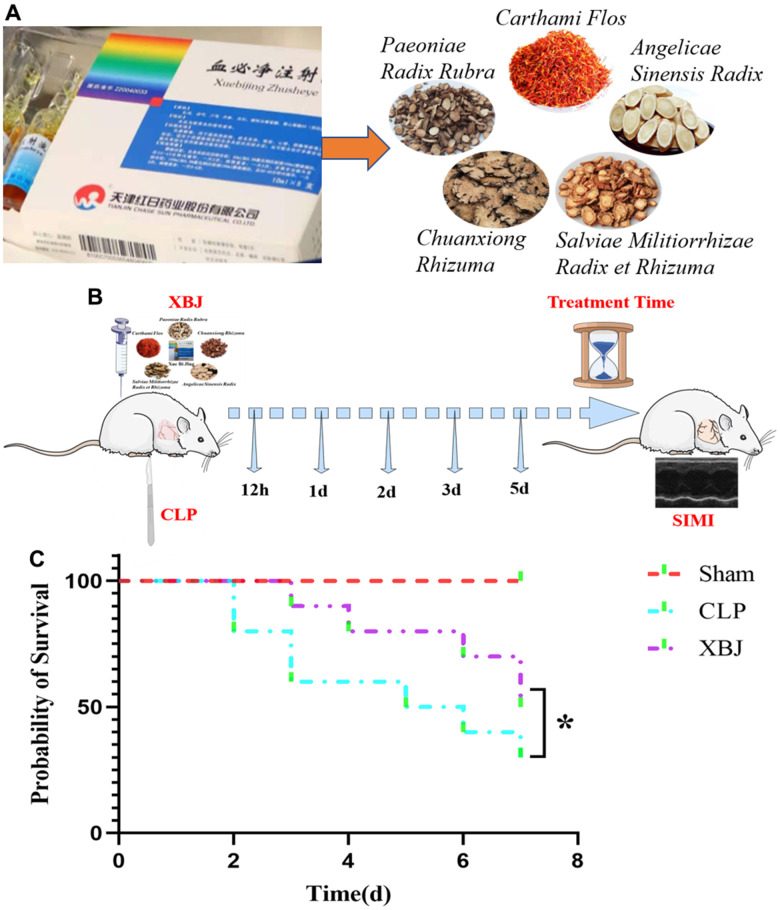
(**A**) The 5 essential drug components of XBJ: *Paeoniae Radix Rubra, Chuanxiong Rhizuma, Angelicae Sinensis Radix, Salviae Militiorrhizae Radix et Rhizuma,* and *Carthami Flos*. (**B**) Graphical Abstract: a guideline of the entire procedure for developing a rat sepsis model and therapeutic administration. (**C**) The survival rate of XBJ for SIMI was evaluated by the Kaplan-Meier method followed by the log rank test within 7 days.

The ingredients in XBJ, a Chinese patent medicine approved for the treatment of sepsis and multi-organ dysfunction in China in 2004, include *Carthamus tinctorius L. (Carthami Flos, hong hua), Paeonia lactiflora Pall. (Paeoniae Radix Rubra, chi shao), Ligusticum chuanxiong Hort. (Chuanxiong Rhizoma, chuan xiong), Salvia miltiorrhiza Bge. (Salviae Miltiorrhizae Radix Et Rhizoma, dan shen)* and *Angelica sinensis (Oliv.) Diels (Angelicae Sinensis Radix, dang gui)* (Figure 1A) [7]. XBJ acts in both the early and late stages of sepsis by anti-inflammatory, anti-coagulation, immune regulation, vascular endothelial protection, anti-oxidative stress and other mechanisms [8]. A key response protein target coverage of 90.16% was found in the target association pattern of the phytoconstituents of XBJ with the causative genes of sepsis, demonstrating the dominancy of XBJ in the treatment of sepsis [9]. However, the underlying therapeutic mechanisms of XBJ on sepsis are still unknown, the specific mechanisms of XBJ in SIMI need to be further investigated.

Sepsis triggers autophagy in multiple organs including the heart [10–12]. More and more studies concentrating on autophagy had shown that the activation of autophagy can improve SIMI [13, 14]. While, some studies have proposed a novel view that the inhibiting of autophagy protects cardiomyocytes from LPS-induced oxidative stress [15, 16], which is in conflict with the previous assertion. What’s more, autophagy triggered inflammatory responses and apoptosis in infected cells [17]. Inflammatory imbalance represents the most critical basis of sepsis pathogenesis and occurs throughout the whole process of sepsis [18]. Inflammation has been center-staged in the field of sepsis research in the past. It is now clear that sepsis is a multifaceted host response to pathogens, involving the early activation of both pro- and anti-inflammatory responses, along with major changes in nonimmunologic pathways [1]. Prior research has suggested that the onset of sepsis was intimately connected to immune paralysis brought on by lymphocyte apoptosis [19]. What’s even more intriguing is that the notion that the majority of sepsis deaths are actually caused by a severely compromised immune response as a result of the widespread apoptosis of immune effector cells is deemed to be more ‘fashionable’ than before [20]. Nevertheless, as the research project of apoptosis in the pathophysiology of sepsis progressed, apoptosis of non-immune cells such as hepatocytes [21], renal cells [22], vascular endothelial cells [23], and cardiomyocytes [13, 14, 24] was also finally discovered. As a consequence, the theory of anti-apoptotic mechanisms underlies a quantity of therapeutic concepts used to treat infectious diseases and inflammatory responses, especially SIMI.

The cell proliferation, autophagy, apoptosis, angiogenesis, epithelial-to-mesenchymal transition, and chemoresistance are all regulated by the PI3K/AKT/mTOR signaling pathway [25]. According to prior study, blocking the PI3K/AKT/mTOR signaling pathway had a protective effect against SIMI; apoptosis that is regulated by PI3K/AKT/mTOR signaling pathway has also been demonstrated to have a significant role in the pathophysiology of SIMI [26]. The study has shown that the PI3K/AKT/mTOR signaling pathway plays an important role in regulating apoptosis and autophagy [27]. Thus, PI3K/AKT/mTOR signaling pathway-regulated apoptosis and autophagy may provide a novel approach to examining XBJ’s action mechanism in the treatment of SIMI.

In this study, a septic rat model was created using CLP, the rats were then given XBJ intraperitoneally, and a series of laboratory tests was conducted. The aim here is to find out how XBJ regulates autophagy and apoptosis mediated by PI3K/AKT/mTOR signaling pathway to enhance SIMI.

## RESULTS

### XBJ increases the survival rate in CLP-induced sepsis rat model

We conducted a survival test in a rat model of sepsis to determine if XBJ has a protective function in CLP-induced sepsis. As shown in Figure 1C, after CLP induction, the survival rate of rats in CLP group began to decline, reaching 80% on 2d, 60% on 3d, 50% on 5d, 40% on 6d and 30% on 7d; whereas, the survival rate of rats in XBJ group started to decrease to 90% on 3d, 80% on 4d, 70% on 6d and 50% on 7d. The rat survival rate in the CLP group was noticeably lower than those in the Sham group (*p* < 0.05), and the administration of 10ml/kg XBJ significantly improves the survival of CLP rat (*p* < 0.05). Our results illustrated that rats exposed to CLP do not develop fatal sepsis within 48 hours and XBJ protected rat from lethal infection with CLP. As a result, this might offer a reasonable starting point for sepsis treatment.

### XBJ attenuates the myocardial dysfunction in CLP-induced sepsis rat model

**Figure 2:**
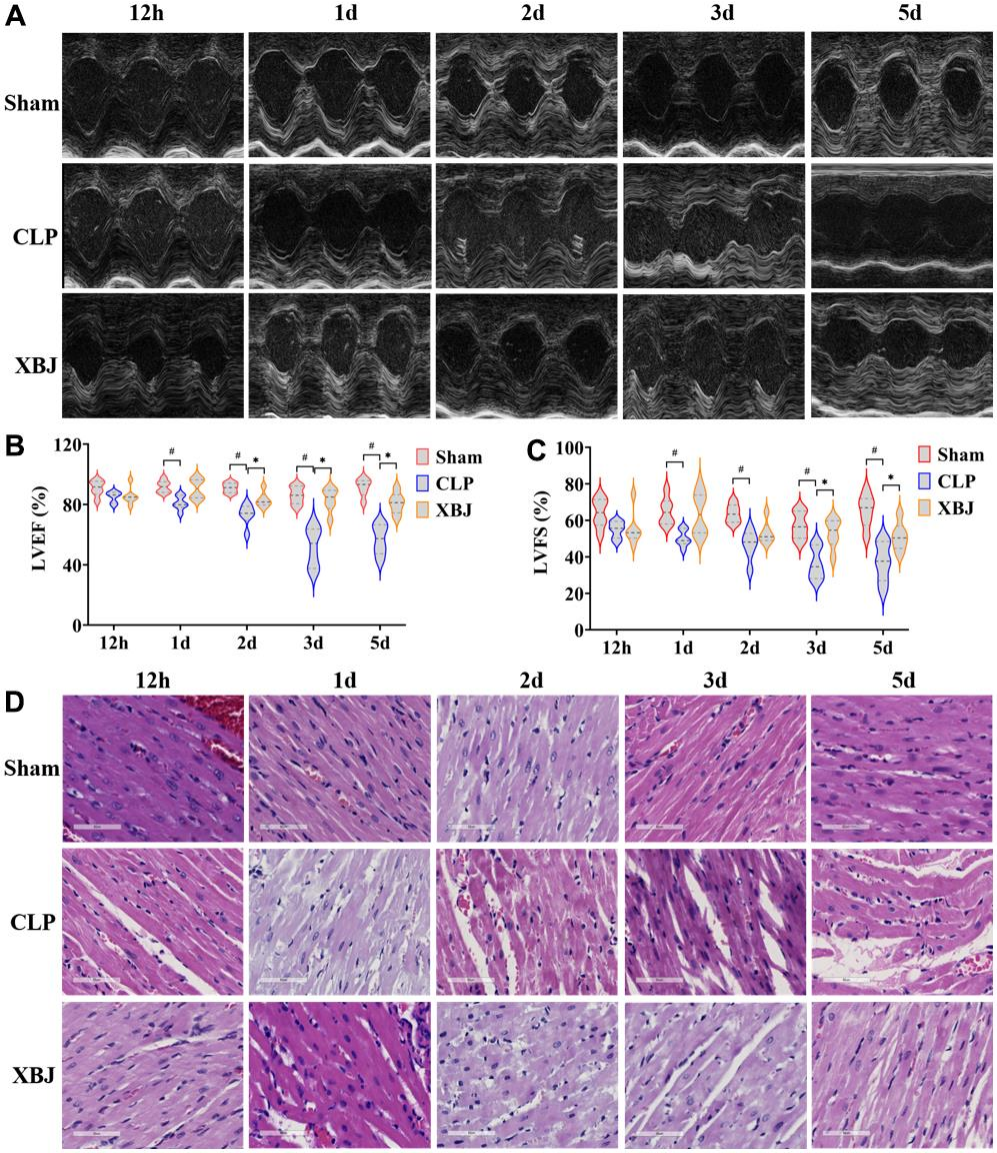
**Effects of XBJ on sepsis-mediated myocardial dysfunction at 12h, 1d, 2d, 3d and 5d after CLP.** (**A**) Representative echocardiographic images. (**B**, **C**) Quantification of LVEF, LVFS via echocardiography. (**D**) Histological analysis of heart via H&E staining (200×).

Rats in the CLP group had dilated hearts, as seen on the echocardiographic images, as opposed to rats in the Sham and XBJ group (Figure 2A). According to Echocardiographic findings, up to 12 hours after CLP induction, the decline in cardiac function in septic rats was not statistically significant (*p* > 0.05) in both the LVEF and LVFS in the CLP group; while there was a significant decrease, which started 1d after CLP (*p* < 0.05). In septic rats, XBJ significantly enhanced LVEF at 2d, 3d, 5d, and LVFS at 3d, 5d (*p* <0.05); however, XBJ’s effect on LVEF at 12h, 1d, and LVFS at 12d, 1d, 2d was not statistically significant (*p* > 0.05, Figure 2B, 2C). Overall, the levels of LVEF, and LVFS dropped in the CLP group while the levels of LVEF, and LVFS increased in the XBJ group in a time-dependent way.

To probe the effect of XBJ on sepsis-induced myocardial injury, CLP-induced rats’ myocardial tissues were detected by H&E staining. As shown in the Figure 2D, H&E staining revealed that as the septic rats’ exposure to CLP was prolonged, the extent of damage to their heart tissue grew progressively more severe. Moreover, the CLP group had noticeable inflammatory cell infiltration compared to the Sham group, interstitial edema, abnormal myocardial cell structure, local necrosis, and indistinct myocardial fiber texture. However, the administration of XBJ obviously ameliorated the foregoing disorder.

These results demonstrated that CLP led to myocardial dysfunction in a time-dependent manner, as well as XBJ improved the myocardial dysfunction in CLP-induced septic rat.

### XBJ improves the myocardial markers and inflammation in CLP-induced sepsis rat model

**Figure 3:**
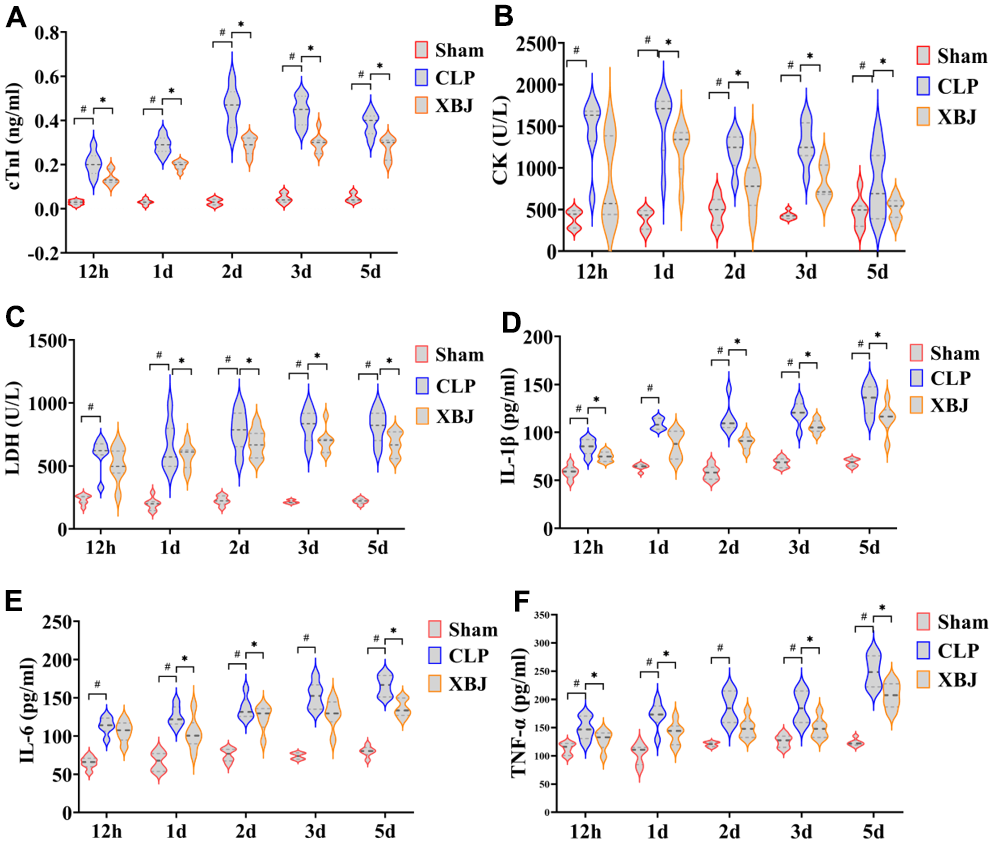
**Effects of XBJ on myocardial markers and serum inflammatory factors at 12h, 1d, 2d, 3d and 5d after CLP.** The levels of serum myocardial markers cTnI (**A**), CK (**B**), and LDH (**C**) were detected by automated biochemical analyzer. The levels of serum inflammatory factors IL-1β (**D**), IL-6 (**E**) and TNF-α (**F**) were detected by ELISA.

We also measured three myocardial injury markers in serum, cTnI, CK, and LDH, using a fully automated biochemical analyzer to visualize myocardial injury in rats. Overall, the cTnI, CK, and LDH levels in the serum were significantly higher in the CLP group than in the Sham group (*p* < 0.05), whereas they were markedly lower in the XBJ group than in the CLP group (*p* < 0.05), with the exception of CK and LDH levels at 12h (*p* > 0.05, Figure 3A–3C). As a whole, the levels of cTnI, CK, and LDH dropped in the XBJ group while the levels of cTnI, CK, and LDH increased in the CLP group in a time-dependent way.

The inflammatory factors the IL-1β, IL-6 and TNF-α levels in the serum were detected by ELISIA. As depicted in Figure 3D–3F, serum inflammatory factors IL-1β, IL-6 and TNF-α levels were significantly higher in the CLP group than in the Sham group (*p* < 0.05), whereas they were markedly lower in the XBJ group than in the CLP group (*p* < 0.05), with the exception of IL-1β at 1d and IL-6 levels at 12h (*p* > 0.05). These results showed as the septic rats’ exposure to CLP was prolonged, the expression level of myocardial inflammatory factors increased in a time-dependent manner, indicating that the degree of myocardial inflammation was gradually aggravated. Additionally, the treatment of XBJ greatly improved the myocardial inflammation in a time-dependent manner.

### XBJ ameliorates the cellular apoptosis of heart tissue in CLP-induced sepsis rat model

**Figure 4:**
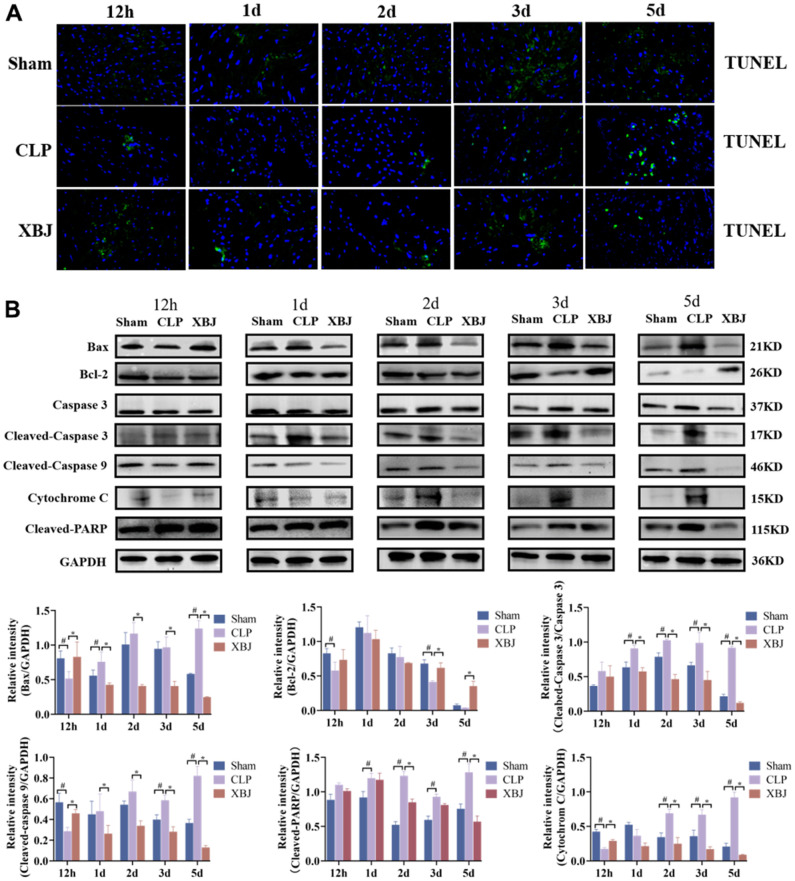
**Effects of XBJ on sepsis-mediated cardiomyocyte apoptosis at 12h, 1d, 2d, 3d and 5d after CLP.** (**A**) Representative images show apoptosis of heart tissue was detected by TUNEL staining. (**B**) Representative images and relative intensity of western blot for Bax, Bcl-2, Caspase 3, Cleaved-Caspase 3, Cleaved-Caspase 9, Cytochrome C and Cleaved-PARP.

We measured the expression of the proteins Bax, Bcl-2, Cleaved-Caspase 3, Cleaved-Caspase 9, Cytochrome C, and Cleaved-PARP by western blot and the apoptotic state by TUNEL staining in the myocardium of different groups over the course of five time points (12h, 1d, 2d, 3d and 5d) to explore the effect of XBJ on cardiomyocyte apoptosis during sepsis. We used TUNEL staining (Figure 4A) to observe that the CLP group’s cardiomyocyte apoptosis was clearly apparent when compared to the myocardial structure of the Sham group at the same point of time. Similarly, as the septic rats’ exposure to CLP was prolonged, the extent of their cardiomyocyte apoptosis grew progressively more severe.

As shown in Figure 4B, the expression of the proteins Bax, Cleaved-Caspase 3, Cleaved-Caspase 9, Cytochrome C, and Cleaved-PARP were significantly increased, whereas the expression of Bcl-2 was significantly decreased in the myocardium of rat with sepsis as compared with the Sham group at the same point of time. Interestingly, treatment with XBJ significantly reversed the foresaid changes. These results indicated that XBJ alleviated SIMI by promoting apoptosis in the early stage of sepsis, and inhibiting apoptosis in the late stage of sepsis.

### XBJ enhances the autophagy of heart tissue in CLP-induced sepsis rat model

**Figure 5:**
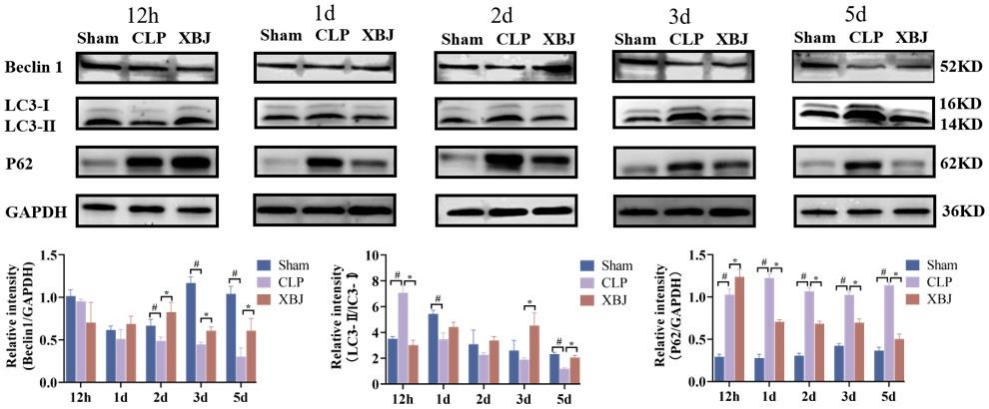
**Effects of XBJ on sepsis-mediated cardiomyocyte autophagy at 12h, 1d, 2d, 3d and 5d after CLP.** Representative images and relative intensity of western blot for Beclin-1, LC3-II/LC3-I and P62.

Autophagosome development is marked by the protein LC3, and the ratio of LC3 II to LC3 I indicates how many autophagosomes are active (positive correlation). Beclin-1 and LC3-II play the vital role to the promotion of autophagy. On the contrary, P62 is regarded as a negative regulator of autophagic activity. To explore the effect of XBJ on cardiomyocyte autophagy during sepsis, we measured LC3-II/LC3-I ratio and the expression of the proteins Beclin-1 and P62 by western blot in the myocardium of different groups over the course of five time points (12h, 1d, 2d, 3d and 5d). As shown in Figure 5, the expression of the protein Beclin-1(except for 12h) and LC3-II/LC3-I ratio (except for 12h) were significantly decreased, whereas the expression of P62 was significantly increased in the CLP group as compared with the Sham group at the same point of time. However, treatment with XBJ significantly augmented the expression level of the protein Beclin-1 (except for 12h) and LC3-II/LC3-I ratio (except for 12h), as well as reduced the expression of P62 (except for 12h) in the myocardium as compared with the CLP group. These results revealed that XBJ alleviated SIMI by inhibiting autophagy in the early stage of sepsis, and enhancing autophagy in the late stage of sepsis.

### XBJ inhibits the PI3K/AKT/mTOR signaling pathway of heart tissue in CLP-induced sepsis rat model

**Figure 6:**
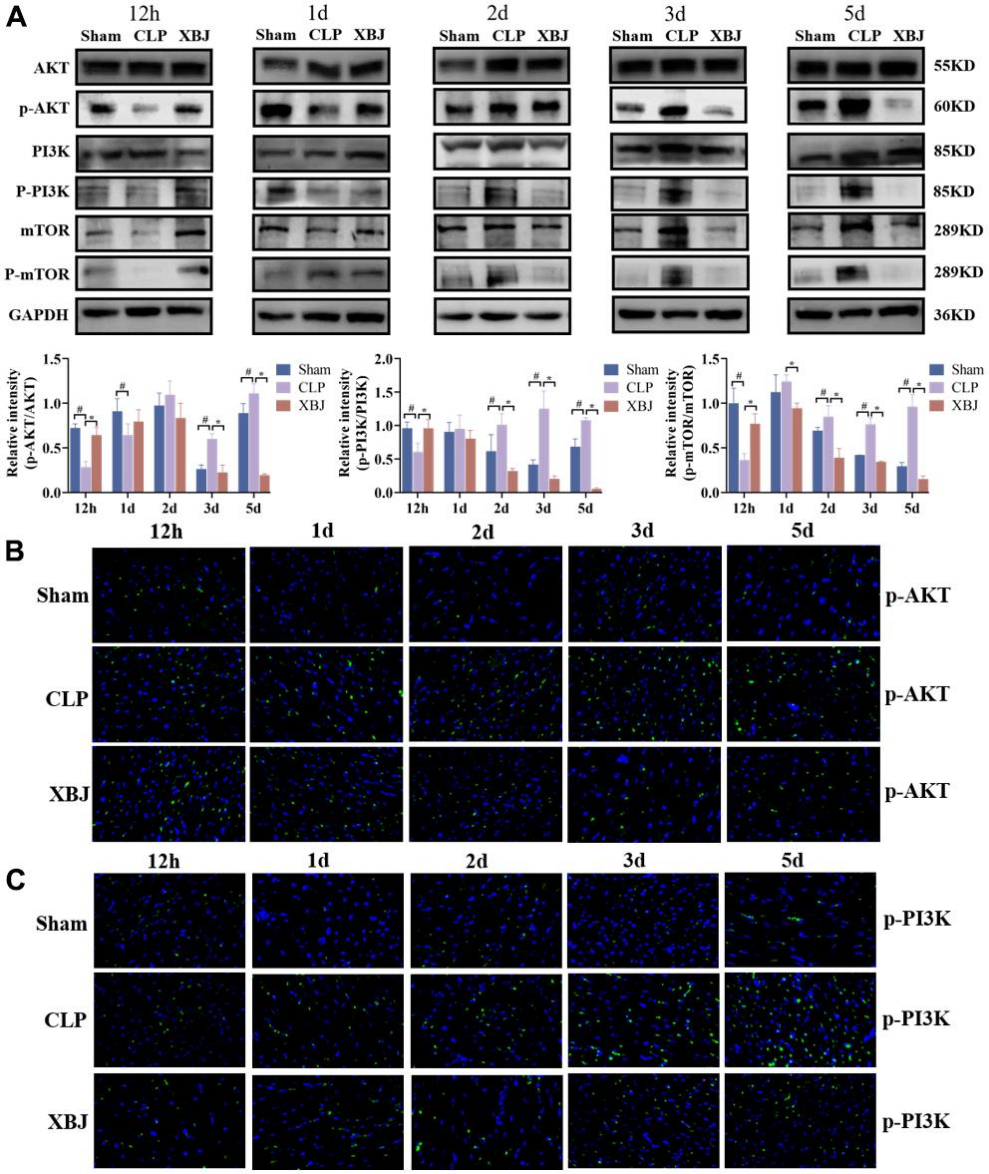
**Effects of XBJ on the PI3K/AKT/mTOR signaling pathway of heart tissue in rat at 12h, 1d, 2d, 3d and 5d after CLP.** (**A**) Representative images and relative intensity of western blot for AKT, p-AKT, PI3K, p-PI3K, mTOR and p-mTOR. (**B**) Representative images of immunofluorescence for p-AKT. (**C**) Representative images of immunofluorescence for p-PI3K.

By measuring the levels of PI3K, AKT, and mTOR in cardiac tissues, the molecular mechanism involving the therapeutic function of XBJ through the regulation of apoptosis and autophagy was further studied. As shown in Figure 6A, the expression of the proteins p-AKT, p-PI3K, and p-mTOR were considerably decreased in the myocardium of the CLP-treated rats at 12h compared to the Sham group, while XBJ dramatically increased these proteins’ levels in CLP-treated rats at this time. Interestingly, the expression of the proteins p-AKT, p-PI3K and p-mTOR were significantly upregulated in the myocardium of rat with sepsis as compared with the Sham group at the same point of time (1d, 2d, 3d and 5d). But, treatment with XBJ significantly downregulated the expression level of the proteins p-AKT, p-PI3K and p-mTOR in the myocardium as compared with the CLP group (1d, 2d, 3d and 5d).

The results of the immunofluorescence assay also demonstrated that the expression of the proteins p-AKT (Figure 6B) and p-PI3K (Figure 6C) were upregulated in the CLP group at the same time as compared to the Sham group, whereas it was downregulated in the XBJ group as compared to the CLP group at the same time. These findings suggested that XBJ can safeguard the myocardium in septic rats by activating the PI3K/AKT /mTOR signaling pathway in the early stages of sepsis and inhibiting it in the latter stages.

## DISCUSSION

**Figure 7:**
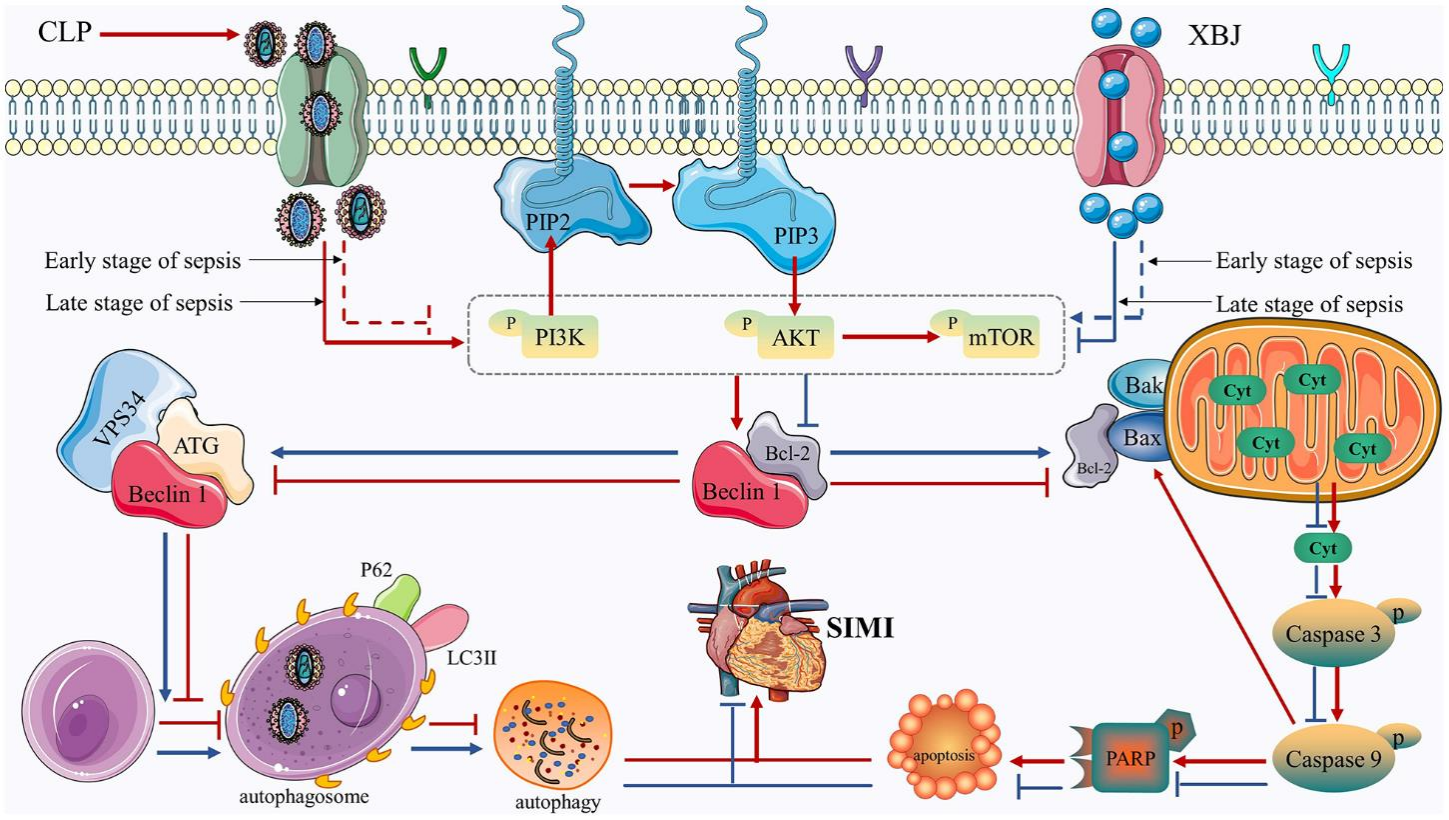
Diagram of the molecular mechanism by which XBJ protects SIMI.

In the present study, we imitated the septic conditions of human using the rat model of CLP and confirmed that XBJ treatment alleviates myocardial injury in rat sepsis models. The mechanism by which XBJ relieves SIMI is to decrease the release of pro-inflammatory cytokines, attenuate cardiomyocyte apoptosis, and enhance autophagy. XBJ exerted these above effects by inhibiting the phosphorylation of the PI3K/AKT/mTOR pathway. In Figure 7, the precise mechanism is displayed.

Early evidence has demonstrated that XBJ administration improved survival rates and protected against myocardial injury in mice models of sepsis [28]. Our results revealed that the 7 days’ mortality of septic rats given XBJ treatment compared favorably to CLP, which is consistent with the previous experiment [29]. In addition, in our study, LVEF and LVFS values were not observed statistically after 12 hour of CLP induction in rats, indicating that cardiac function may not be unimpaired, which may be the result of the compensatory state of the heart in the early stage of sepsis, while it was significantly reduced after 1 day (Figure 2A–2C); H&E staining (Figure 2D) showed that the degree of cardiac tissue damage induced by CLP was serious; the serum levels of myocardial injury markers cTnI, CK, and LDH (Figure 3A–3C) were significantly increased in CLP-induced rats. The results showed the rat model of sepsis was successfully established using CLP. Meanwhile, a study showed that XBJ attenuated the myocardial dysfunction/injury and systemic inflammatory response of the CLP-induced septic rat [30]. Another study found that XBJ drastically enhanced cardiac function in septic mice in 24h after CLP [30]. In our study, the outcomes of echocardiography, H&E staining and the serum levels of myocardial injury markers showed that XBJ improved significantly cardiac dysfunction in septic rats after CLP.

What’s more, sepsis induces systemic inflammation [31]. It is known from the current literature [32, 33] that two pathophysiological processes—exogenous pathogen-associated molecular patterns (PAMPs) and endogenous damage-associated molecular patterns (DAMPs)—are closely related to the development of sepsis. During sepsis, the pathogen-associated molecular patterns trigger systemic inflammatory responses, leading to an excess production of proinflammatory cytokines that are responsible for coagulopathy, such as interleukin IL-1β, IL-6, and tumor necrosis factor-α (TNF-α) [34]. Studies have shown inflammation in multiple organs in experimental sepsis induced by LPS or CLP, including the heart, the kidney, the liver, and the lung [35–38]. Researchers have also consistently observed elevated circulating levels of proinflammatory cytokines such as IL-1β, IL-6, and TNF-α in septic patients [39]. It has been reported that pretreatment with XBJ reduces the serum levels of TNF-α and IL-6 in a dose-dependent manner [40]. In addition, it has been also reported that XBJ effectively reduced circulating IL-1β, IL-6, and TNF-α in CLP rats within 12 h after sepsis [41]. In our study, the plasma levels of proinflammatory cytokines IL-1β, IL-6 and TNF-α were markedly elevated in CLP group rats at 12 hours to 5 days, also demonstrating once again the successful establishment of a CLP-induced sepsis model. However, treatment with XBJ significantly reduced these inflammatory factor levels (Figure 3D–3F), suggesting that XBJ administration alleviates sepsis-induced systemic inflammation in CLP rats.

Apoptosis that is caused by an excessive inflammatory response plays an essential role in SIMI as well as the pathophysiological process that leads to sepsis [42]. Apoptosis is a tightly regulated form of cell death that is vital in both embryo implantation and development and turnover of tissues during maturation [43]. During sepsis-induced immunosuppression, apoptosis plays a pivotal role in tissues/organs [44]. Numerous fundamental investigations have shown that lowering cardiomyocyte apoptosis can protect against cardiac dysfunction in sepsis [45, 46]. Additionally, the key biological functional modules including cell apoptosis of XBJ for treating sepsis were identified [30]. By examining the levels of apoptosis-related gene expression in sepsis patients, Weber SU et al. revealed that apoptosis was sparked in the early stages of the condition [47]. In our result, TUNEL staining (Figure 4A) proved that XBJ dramatically reduced cardiomyocyte apoptosis in the myocardium as likened to the CLP group. Previous studies have found that CLP induced the over-expression of Bax, Cleaved-Caspase 3, Cleaved-Caspase 9 and Cytochrome C and the low-expression of Bcl-2 in rat heart tissue [14, 48, 49]. Our current findings indicate that the myocardium in sepsis rat does not express more pro-apoptotic genes in response to stimulation during the first 12 hours of CLP exposure. The explanation for this may be that when rats were first exposed to CLP, Bax was unable to trigger the opening of mitochondrial outer membrane permeability and was unable to stimulate a variety of pro-apoptotic proteins, which lead to anti-apoptotic proteins predominated in the body. Generally speaking, XBJ reduced the expression of pro-apoptotic proteins and increased the expression of anti-apoptotic proteins during the early 12h of CLP-induced sepsis, while these indexes were reversed during the late 1 day to 5 days (Figure 4B). These results showed that XBJ alleviated SIMI by promoting apoptosis in the early stage of sepsis, and inhibiting apoptosis in the late stage of sepsis.

Autophagy is one of the mechanisms involved in the pathophysiology of SIMI, and a homeostasis process involving self-digestion for energy [50]. Autophagy contributes to the preventive function during infectious diseases [51]. Autophagosome formation and maturation are regulated by several core autophagy gene (ATG) proteins in a highly controlled manner. Beclin-1 is a mammalian cell homolog of the yeast autophagy-related gene ATG6, and mediates the initiation stage of autophagy [52]. A study showed that injection of a cell-permeable Tat-Beclin-1 peptide to activate autophagy improved cardiac function, attenuated inflammation, and rescued the phenotypes caused by Beclin-1 deficiency in LPS-challenged mice [35]. Microtubule-associated protein LC3, another key protein in autophagy, is encoded by a mammalian ortholog of yeast Atg8 that is ubiquitous in mammalian tissues and cultured cells. It has two forms, LC3-I and LC3-II [53]. The autophagic vesicle membrane is where LC3-II is located after LC3-I is typically transformed to LC3-II during the development of an autophagosome [54]. P62 functions in autophagy as a selective junction protein by modifying ubiquitinated proteins and delivering them to the proteasome for destruction [55]. However, whether autophagy is inhibited or activated throughout the pathophysiological process of sepsis is still a topic of debate. A study showed that the pathophysiological process of sepsis’ organ failure is mostly due to blocked autophagy [11]. Other studies showed increased autophagy in the early phases of sepsis followed by a decline near late-stage organ failure in the mouse CLP sepsis model [56, 57]. Several basic studies presenting various therapeutic methods have determined that SIMI can be improved by mediating autophagy [13, 58, 59]. Since autophagy is a dynamic and intricate process, it is challenging for us to record consistent and uniform autophagic changes over the course of numerous static time periods. Therefore, the dynamic time changes (1h, 1d, 2d, 3d and 5d) during sepsis were investigated in the present study to clarify the mechanism of SIMI. The results showed that compared with the CLP group, Beclin-1 expression was low, LC3-II/LC3-I ratio decreased, and P62 accumulation was evident at 12 h of XBJ intervention, and all data were reversed at 1 to 5 days (Figure 5), demonstrated XBJ alleviated SIMI by inhibiting autophagy in the early stage of sepsis, and enhancing autophagy in the late stage of sepsis.

We will further investigate XBJ’s impact on the PI3K/AKT/mTOR pathway after identifying its cardioprotective role of regulating apoptosis and autophagy during sepsis. Regulation of PI3K/AKT/mTOR pathway is mediated in the mechanism of apoptosis and autophagy, which has been confirmed in animal experiments [60, 61]. According to a recent systemic pharmacological analysis, XBJ can affect sepsis by acting on alterations in a number of genes involved in the PI3K/AKT/mTOR signaling pathway [8, 9]. Previous research has found that when applied to sepsis, XBJ blocked the phosphorylation of the PI3K/AKT/mTOR signaling pathway [62]. However, there have been two divergent voices on the road to sepsis mitigation research. The first one is that the improvement of SIMI is through the activation of PI3K/AKT/mTOR signaling pathway [63, 64], which is in line with our findings at 12h that administration of XBJ therapy increased the phosphorylation levels of PI3K, AKT and mTOR in CLP-induced sepsis, suggesting by activation of the PI3K/AKT/mTOR pathway. Study effectively inhibited hypoxia and reoxygenation-induced autophagy and apoptosis via, at least partially, activating the PI3K/AKT/mTOR pathways. The second one is that blocking the PI3K/AKT/mTOR signaling pathway had a protective effect against sepsis [26, 27], which is in line with our findings at 1d, 2d, 3d and 5d that administration of XBJ therapy suppressed p-PI3K, p-AKT and p-mTOR, showing by inhibition of the PI3K/AKT/mTOR pathway. Meanwhile, the PI3K/AKT/mTOR signaling pathway was blocked during sepsis, to trigger autophagy and block apoptosis [27]. Based on our results and previous literature we speculated that XBJ can safeguard the myocardium in septic rats by activating the PI3K/AKT /mTOR signaling pathway in the early stages of sepsis and inhibiting it in the latter stages. Therefore, we speculated that in CLP-induced septic rats, XBJ implementation may potentially result in inhibiting apoptosis and promoting autophagy via activating PI3K/AKT/mTOR pathway in the early stage of sepsis, as well as promoting apoptosis and inhibiting autophagy via, at least partially, suppressing PI3K/AKT/mTOR pathway in the late stage of sepsis. The specific mechanism of this regulation still needs to be proved by further studies. Figure 7 presents a diagram of the molecular mechanism by which XBJ protects SIMI.

The innovative point of the present study is the fact that we utilized five time points to monitor the long-term changing regularity of SIMI and XBJ’s myocardial protective mechanisms in sepsis, which is more in accord with the dynamic and intricate features of sepsis. Although there are important discoveries revealed by these studies, there are also limitations. First, the precise molecular mechanism by which XBJ affects the PI3K/AKT/mTOR pathway in sepsis remains unknown. Second, we have to point out that we do not look into how changes to the PI3K/AKT/mTOR pathway XBJ work to affect cardiomyocyte apoptosis and autophagy during sepsis in further detail.

## CONCLUSIONS

Here, we presented strong support for a putative mechanism through which XBJ defends against SIMI caused by CLP. It has been found that CLP induced myocardial injury and dysfunction at a time-dependent manner. Additionally, based on our results, the possible molecular mechanism of XBJ in the treatment of CLP-induced SIMI speculated that XBJ’s cardioprotective advantages may be attributable to its ability to suppress apoptosis and promote autophagy via, at least partially, activating PI3K/AKT/mTOR pathway in the early stage of sepsis, as well as promoting apoptosis and inhibit autophagy via suppressing PI3K/AKT/mTOR pathway in the late stage of sepsis.

## MATERIALS AND METHODS

### Animal experiment

All animal procedures were carried out in accordance with the regulations of the Animal Protection Committee of Ningxia Medical University, and all experimental procedures were approved by the Ethics Committee of the General Hospital of Ningxia Medical University. 6-8 weeks male specific pathogen free Sprague-Dawley rats (220 ± 20 g), were purchased from and housed at the Ningxia Medical University laboratory animal center. Rats were raised in plastic cages with temperature and humidity-controlled room (22.8±2.0° C and 50%~60%, respectively) with a 12/12 hours light/dark cycle and allowed ad libitum access to food and water.

### Polymicrobial sepsis caused by CLP

Polymicrobial sepsis was generated in rat by CLP surgery as previously described [65]. To put it simply, the rats were fixed on the operating table after anesthesia (4% phenobarbital (40 mg/kg i.p.)), the abdominal hair was removed for routine disinfection, the abdominal cavity was opened to expose the cecum, and the distal 50% was ligation. A “penetrating” operation with 22-gauge needle was then performed on the ligation of the cecum. Abdominal incision and intestinal manipulation with neither ligation nor puncture were performed in sham-operated animals. All rats were subcutaneous injected with lactated Ringer’s solution (30 ml/kg) after injury for fluid resuscitation and housed alone. Postoperative pain was managed by antibiotic Imipenem/Cilastin (20 mg/kg s.c.) and analgesia flurbiprofen axetil injection (5 mg/kg i.v.).

### Drug treatment and sample collection

Rats were randomly divided into 3 groups: (1) Sham group; (2) CLP group; and (3) XBJ group. Three groups of rats after Sham or CLP were randomly divided into 12 hours, 1 day, 2 days, 3 days and 5 days groups (n = 6/group). The animals in each group were treated with drugs for 12 hours, 1 day, 2 days, 3 days or 5 days, respectively. One hour after CLP, rats in XBJ group were treated with XBJ (10 ml/kg s.c.), while rats in sham or CLP group were treated with the same volume of normal saline. See Figure 1B for details.

Animals under anesthesia were used for echocardiographic evaluation. Blood samples in heparinized tubes were collected from the heart, centrifuged at 3500 rpm/min for 10 min at room temperature, and the supernatant serum was aspirated and stored at -80° C for the subsequent analyses. Then, the rats were euthanized by excessive anesthesia administration. The heart tissues were stored in 4% paraformaldehyde at 4° C for Hematoxylin and Eosin (H&E) staining, TdT mediated dUTP biotin nick-end labeling (TUNEL) staining and Immunofluorescence assay, and cryopreserved in liquid nitrogen for protein western blot.

### Survival curve

Another thirty rats were also subjected to the CLP or sham procedure as mentioned above to investigate survival. After the CLP or sham surgery, the survival of the rat was observed every 12 h up to 7 days.

### Echocardiography

Cardiac function was assessed using an Ultra High Resolution Small Animal Ultrasound Imaging System (Vevo®2100 Imaging System, Visualsonics, Toronto, Canada) with a 15-MHz transducer. After the induction of general anaesthesia with 4% pentobarbital (40 mg/kg i.p.), hearts were imaged in 2-D mode in the parasternal long-axis view prior to M-mode imaging positioned perpendicular to the interventricular septum and posterior left ventricular wall. Heart rate was measured over 3 consecutive cycles. The left ventricular ejection fraction (LVEF) and left ventricular fractional shortening (LVFS) parameters were calculated by the software of Vevo770TM imaging system.

### Biochemical detection

The enzyme activity of cardiac troponin I (cTnI), creatine kinase (CK), lactate dehydrogenase (LDH) levels in serum was measured using a quick, convenient, and sensitive corresponding assay kit based on the protocol. Serum concentrations of these factors were detected by an Automatic Biochemical Analyzer (Bio Majesty JCA-BM6010, JEOL Ltd., Japan).

### Enzyme-linked immunosorbent assay (ELISA)

Inflammatory cytokines, interleukin-1β (IL-1β), IL-6 and tumor necrosis factor-α (TNF-α) in serum were measured using ELISA kits, according to the manufacturer’s instructions (BioSwamp, Wuhan, China). The concentrations of the cytokines were quantified by referring to standard curves.

### Histopathological (H&E) staining

4 μm sections were stained using a H&E Staining Kit (Biotopped, Beijing, China), according to the manufacturer’s instructions. Morphological changes in myocardial tissues were observed by H&E under a light microscope. Morphological changes in myocardial tissues were observed at 200 × magnification under a light microscope (Leica, USA). Three hearts were analyzed per group.

### TUNEL staining

Extensive DNA degradation is the signature of the late stage of apoptosis. Visualization of apoptotic cardiomyocytes was performed on left ventricular tissue cross sections (4 μm thick) using One-step TUNEL Apoptosis Detection Kit (Beyotime, Beijing, China) and according to the manufacturer’s procedure. TUNEL staining changes in myocardial tissues were observed with a fluorescence microscope (MF43-N, Mshot, China) to obtain representative fluorescence images.

### Immunofluorescence assay

Immunofluorescence was used to assess the level of p-AKT (Ser473) and p-PI3K (Tyr607) in heart tissue. Fixed heart tissues were removed with 0.5% Triton X-100 for 20 min. Tissues were blocked with 5% BSA blocking solution for 60 min at room temperature, following by washing with PBS. The tissues were then incubated with p-AKT (Ser473) antibody (cat. AF0016,1:500) and p-PI3K (Tyr607) antibody (cat. AF3241, 1:500) overnight at 4° C and further stained with Goat anti-Rabbit lgG H&L (AlexaFluor®594) (cat. ZF-0516, 1:100) secondary antibody. Afterwards, heart tissue was stained with 4′, 6-diamidino-2-phenylindole (DAPI, C0060, Solarbio, China) and observed with a fluorescence microscope (MF43-N, Mshot, China) to obtain representative fluorescence images.

### Western blot

The heart tissues added to RIPA lysis buffer spiked with protease inhibitors and phosphorylated protease inhibitors (Servicebio, Wuhan, China) are crushed by adding magnetic beads in Fully automatic sample freezer grinder (JXFSTPRP-CL, Shanghai Jing Xin, China). The protein concentration was measured using a BCA protein assay kit (Omni-Easy, Shanghai, China). Equal amounts of protein (5 μg/μl, 10 μl per lane) were separated by 7.5-12.5% SDS-PAGE and were transferred onto PVDF membranes using Bio-Rad western blot analysis apparatus (CAVOY, Beijing, China). The membranes were then blocked with 5% skim milk powder at room temperature for 2 h and incubated at 4° C overnight with antibodies against PI3K (cat. AF6241, 1:1000), phosphorylated (p)-PI3K (cat. AF3241, 1:1000), AKT (cat. AF6261, 1:1000), p-AKT (cat. AF0016, 1:1000), mTOR (cat. AF6308, 1:1500), p-mTOR (cat. AF3308, 1:1500), Bax (cat. AF0120, 1:2000), Bcl-2 (cat. AF6139, 1:2000), Cleaved-caspase 3 (cat. AF7022, 1:1000), Caspase 3 (cat. AF6311, 1:1000), Cleaved-caspase 9 (cat. AF5240, 1:1000), Cleaved-PARP (cat. AF7023, 1:1000), Cytochrome C (cat. AF0146, 1:1000), GAPDH (cat. T0004; 1:10,000), P62 (cat. Ab91526, 1:1000), Beclin 1 (cat. Ab62557, 1:1000) or LC3-I/II (cat. Ab128025, 1:1000), followed by incubation at room temperature for 1h with goat anti-rabbit secondary antibodies (cat. S001; 1:10,000) or goat anti-mouse secondary antibodies (cat. AS014; 1:10,000; Abclonal). GAPDH was used as the internal reference protein. Protein bands were detected with an enhanced chemiluminescence kit (KeyGen BioTECH, Jiangshu, China) using capturing light sources with an ultrasensitive multifunction imager (Amersham lmager 680RGB) and were semi-quantified using ImageJ software (Rawak Software, Inc. Germany).

### Statistical analysis

All values described in the text and figures are presented as mean ± standard deviation (SD). The Kaplan-Meier method was applied to assess survival followed by the log rank test. One-way analysis of variance (ANOVA) test was used to compare among multiple groups, followed by Tukey’s test after a homogeneity test for variance and Tamhane T2’s test after a heterogeneity test for variance. SPSS 24.0 software was used to analyze the data. *p* < 0.05 in two-tailed testing was considered statistically significant. In all the resulting graphs, # indicates *p* < 0.05 (vs. the Sham group) and * indicates *p* < 0.05 (vs. the CLP group).

## References

[1] Seymour CW, Liu VX, Iwashyna TJ, Brunkhorst FM, Rea TD, Scherag A, Rubenfeld G, Kahn JM, Shankar-Hari M, Singer M, Deutschman CS, Escobar GJ, Angus DC. Assessment of Clinical Criteria for Sepsis: For the Third International Consensus Definitions for Sepsis and Septic Shock (Sepsis-3). JAMA. 2016; 315:762–74. 10.1001/jama.2016.0288 26903335 PMC5433435

[2] Rudd KE, Johnson SC, Agesa KM, Shackelford KA, Tsoi D, Kievlan DR, Colombara DV, Ikuta KS, Kissoon N, Finfer S, Fleischmann-Struzek C, Machado FR, Reinhart KK, et al. Global, regional, and national sepsis incidence and mortality, 1990-2017: analysis for the Global Burden of Disease Study. Lancet. 2020; 395:200–11. 10.1016/S0140-6736(19)32989-7 31954465 PMC6970225

[3] Markwart R, Saito H, Harder T, Tomczyk S, Cassini A, Fleischmann-Struzek C, Reichert F, Eckmanns T, Allegranzi B. Epidemiology and burden of sepsis acquired in hospitals and intensive care units: a systematic review and meta-analysis. Intensive Care Med. 2020; 46:1536–51. 10.1007/s00134-020-06106-2 32591853 PMC7381455

[4] Hollenberg SM, Singer M. Pathophysiology of sepsis-induced cardiomyopathy. Nat Rev Cardiol. 2021; 18:424–34. 10.1038/s41569-020-00492-2 33473203

[5] Lakshmikanth CL, Jacob SP, Chaithra VH, de Castro-Faria-Neto HC, Marathe GK. Sepsis: in search of cure. Inflamm Res. 2016; 65:587–602. 10.1007/s00011-016-0937-y 26995266

[6] Lelubre C, Vincent JL. Mechanisms and treatment of organ failure in sepsis. Nat Rev Nephrol. 2018; 14:417–27. 10.1038/s41581-018-0005-7 29691495

[7] Li C, Wang P, Zhang L, Li M, Lei X, Liu S, Feng Z, Yao Y, Chang B, Liu B, Shang H. Efficacy and safety of Xuebijing injection (a Chinese patent) for sepsis: A meta-analysis of randomized controlled trials. J Ethnopharmacol. 2018; 224:512–21. 10.1016/j.jep.2018.05.043 29860133

[8] Li C, Wang P, Li M, Zheng R, Chen S, Liu S, Feng Z, Yao Y, Shang H. The current evidence for the treatment of sepsis with Xuebijing injection: Bioactive constituents, findings of clinical studies and potential mechanisms. J Ethnopharmacol. 2021; 265:113301. 10.1016/j.jep.2020.113301 32860891

[9] Wu Q, Yin CH, Li Y, Cai JQ, Yang HY, Huang YY, Zheng YX, Xiong K, Yu HL, Lu AP, Wang KX, Guan DG, Chen YP. Detecting Critical Functional Ingredients Group and Mechanism of Xuebijing Injection in Treating Sepsis. Front Pharmacol. 2021; 12:769190. 10.3389/fphar.2021.769190 34938184 PMC8687625

[10] Sun Y, Cai Y, Zang QS. Cardiac Autophagy in Sepsis. Cells. 2019; 8:141. 10.3390/cells8020141 30744190 PMC6406743

[11] Liu Q, Wu J, Zhang X, Li X, Wu X, Zhao Y, Ren J. Circulating mitochondrial DNA-triggered autophagy dysfunction via STING underlies sepsis-related acute lung injury. Cell Death Dis. 2021; 12:673. 10.1038/s41419-021-03961-9 34218252 PMC8254453

[12] Deng Z, Sun M, Wu J, Fang H, Cai S, An S, Huang Q, Chen Z, Wu C, Zhou Z, Hu H, Zeng Z. SIRT1 attenuates sepsis-induced acute kidney injury via Beclin1 deacetylation-mediated autophagy activation. Cell Death Dis. 2021; 12:217. 10.1038/s41419-021-03508-y 33637691 PMC7910451

[13] Zhang WX, He BM, Wu Y, Qiao JF, Peng ZY. Melatonin protects against sepsis-induced cardiac dysfunction by regulating apoptosis and autophagy via activation of SIRT1 in mice. Life Sci. 2019; 217:8–15. 10.1016/j.lfs.2018.11.055 30500551

[14] Wang X, Xie D, Dai H, Ye J, Liu Y, Fei A. Clemastine protects against sepsis-induced myocardial injury *in vivo* and *in vitro*. Bioengineered. 2022; 13:7134–46. 10.1080/21655979.2022.2047256 35274595 PMC9208445

[15] Lei S, Zhang Y, Su W, Zhou L, Xu J, Xia ZY. Remifentanil attenuates lipopolysaccharide-induced oxidative injury by downregulating PKCβ2 activation and inhibiting autophagy in H9C2 cardiomyocytes. Life Sci. 2018; 213:109–15. 10.1016/j.lfs.2018.10.041 30352239

[16] Yang Z, Su W, Zhang Y, Zhou L, Xia ZY, Lei S. Selective inhibition of PKCβ2 improves Caveolin-3/eNOS signaling and attenuates lipopolysaccharide-induced injury by inhibiting autophagy in H9C2 cardiomyocytes. J Mol Histol. 2021; 52:705–15. 10.1007/s10735-021-09990-0 34105058

[17] Li F, Li J, Wang PH, Yang N, Huang J, Ou J, Xu T, Zhao X, Liu T, Huang X, Wang Q, Li M, Yang L, et al. SARS-CoV-2 spike promotes inflammation and apoptosis through autophagy by ROS-suppressed PI3K/AKT/mTOR signaling. Biochim Biophys Acta Mol Basis Dis. 2021; 1867:166260. 10.1016/j.bbadis.2021.166260 34461258 PMC8390448

[18] Grondman I, Pirvu A, Riza A, Ioana M, Netea MG. Biomarkers of inflammation and the etiology of sepsis. Biochem Soc Trans. 2020; 48:1–14. 10.1042/BST20190029 32049312

[19] Lang JD, Matute-Bello G. Lymphocytes, apoptosis and sepsis: making the jump from mice to humans. Crit Care. 2009; 13:109. 10.1186/cc7144 19216722 PMC2688100

[20] Hotchkiss RS, Nicholson DW. Apoptosis and caspases regulate death and inflammation in sepsis. Nat Rev Immunol. 2006; 6:813–22. 10.1038/nri1943 17039247

[21] Wu Y, Zhao M, Lin Z. Pyrroloquinoline quinone (PQQ) alleviated sepsis-induced acute liver injury, inflammation, oxidative stress and cell apoptosis by downregulating CUL3 expression. Bioengineered. 2021; 12:2459–68. 10.1080/21655979.2021.1935136 34227919 PMC8806920

[22] Lu Y, Yang Y, He X, Dong S, Wang W, Wang D, Zhang P. Esmolol reduces apoptosis and inflammation in early sepsis rats with abdominal infection. Am J Emerg Med. 2017; 35:1480–4. 10.1016/j.ajem.2017.04.056 28457762

[23] Zhou M, Simms HH, Wang P. Adrenomedullin and adrenomedullin binding protein-1 attenuate vascular endothelial cell apoptosis in sepsis. Ann Surg. 2004; 240:321–30. 10.1097/01.sla.0000133253.45591.5b 15273558 PMC1356410

[24] Li Z, Meng Y, Liu C, Liu H, Cao W, Tong C, Lu M, Li L, Peng L. Kcnh2 mediates FAK/AKT-FOXO3A pathway to attenuate sepsis-induced cardiac dysfunction. Cell Prolif. 2021; 54:e12962. 10.1111/cpr.12962 33263944 PMC7848965

[25] Peng Y, Wang Y, Zhou C, Mei W, Zeng C. PI3K/Akt/mTOR Pathway and Its Role in Cancer Therapeutics: Are We Making Headway? Front Oncol. 2022; 12:819128. 10.3389/fonc.2022.819128 35402264 PMC8987494

[26] Xie WJ, Hou G, Wang L, Wang SS, Xiong XX. Astaxanthin suppresses lipopolysaccharide-induced myocardial injury by regulating MAPK and PI3K/AKT/mTOR/GSK3β signaling. Mol Med Rep. 2020; 22:3338–46. 10.3892/mmr.2020.11443 32945516 PMC7453592

[27] Zhao Y, Feng X, Li B, Sha J, Wang C, Yang T, Cui H, Fan H. Dexmedetomidine Protects Against Lipopolysaccharide-Induced Acute Kidney Injury by Enhancing Autophagy Through Inhibition of the PI3K/AKT/mTOR Pathway. Front Pharmacol. 2020; 11:128. 10.3389/fphar.2020.00128 32158395 PMC7052304

[28] Cao L, Li Z, Ren Y, Wang M, Yang Z, Zhang W, Han X, Yao M, Sun Z, Nie S. Xuebijing Protects Against Septic Acute Liver Injury Based on Regulation of GSK-3β Pathway. Front Pharmacol. 2021; 12:627716. 10.3389/fphar.2021.627716 33995024 PMC8120308

[29] Jiang Y, Zou L, Liu S, Liu X, Chen F, Liu X, Zhu Y. GC/MS-based metabonomics approach reveals effects of Xuebijing injection in CLP induced septic rats. Biomed Pharmacother. 2019; 117:109163. 10.1016/j.biopha.2019.109163 31238257

[30] Zhou W, Lai X, Wang X, Yao X, Wang W, Li S. Network pharmacology to explore the anti-inflammatory mechanism of Xuebijing in the treatment of sepsis. Phytomedicine. 2021; 85:153543. 10.1016/j.phymed.2021.153543 33799226

[31] Nedeva C, Menassa J, Puthalakath H. Sepsis: Inflammation Is a Necessary Evil. Front Cell Dev Biol. 2019; 7:108. 10.3389/fcell.2019.00108 31281814 PMC6596337

[32] Gong T, Liu L, Jiang W, Zhou R. DAMP-sensing receptors in sterile inflammation and inflammatory diseases. Nat Rev Immunol. 2020; 20:95–112. 10.1038/s41577-019-0215-7 31558839

[33] Gotts JE, Matthay MA. Sepsis: pathophysiology and clinical management. BMJ. 2016; 353:i1585. 10.1136/bmj.i1585 27217054

[34] Lupu F, Keshari RS, Lambris JD, Coggeshall KM. Crosstalk between the coagulation and complement systems in sepsis. Thromb Res. 2014 (Suppl 1); 133:S28–31. 10.1016/j.thromres.2014.03.014 24759136 PMC4154483

[35] Sun Y, Yao X, Zhang QJ, Zhu M, Liu ZP, Ci B, Xie Y, Carlson D, Rothermel BA, Sun Y, Levine B, Hill JA, Wolf SE, et al. Beclin-1-Dependent Autophagy Protects the Heart During Sepsis. Circulation. 2018; 138:2247–62. 10.1161/CIRCULATIONAHA.117.032821 29853517 PMC6274625

[36] Xu C, Chang A, Hack BK, Eadon MT, Alper SL, Cunningham PN. TNF-mediated damage to glomerular endothelium is an important determinant of acute kidney injury in sepsis. Kidney Int. 2014; 85:72–81. 10.1038/ki.2013.286 23903370 PMC3834073

[37] Ito Y, Abril ER, Bethea NW, McCuskey MK, Cover C, Jaeschke H, McCuskey RS. Mechanisms and pathophysiological implications of sinusoidal endothelial cell gap formation following treatment with galactosamine/endotoxin in mice. Am J Physiol Gastrointest Liver Physiol. 2006; 291:G211–8. 10.1152/ajpgi.00312.2005 16574994

[38] Schmidt EP, Yang Y, Janssen WJ, Gandjeva A, Perez MJ, Barthel L, Zemans RL, Bowman JC, Koyanagi DE, Yunt ZX, Smith LP, Cheng SS, Overdier KH, et al. The pulmonary endothelial glycocalyx regulates neutrophil adhesion and lung injury during experimental sepsis. Nat Med. 2012; 18:1217–23. 10.1038/nm.2843 22820644 PMC3723751

[39] Wang L, Zhao H, Wang D. Inflammatory cytokine expression in patients with sepsis at an intensive care unit. Exp Ther Med. 2018; 16:2126–31. 10.3892/etm.2018.6376 30186449 PMC6122406

[40] Xu Q, Liu J, Guo X, Tang Y, Zhou G, Liu Y, Huang Q, Geng Y, Liu Z, Su L. Xuebijing injection reduces organ injuries and improves survival by attenuating inflammatory responses and endothelial injury in heatstroke mice. BMC Complement Altern Med. 2015; 15:4. 10.1186/s12906-015-0519-5 25653103 PMC4323134

[41] Lv J, Guo X, Zhao H, Zhou G, An Y. Xuebijing Administration Alleviates Pulmonary Endothelial Inflammation and Coagulation Dysregulation in the Early Phase of Sepsis in Rats. J Clin Med. 2022; 11:6696. 10.3390/jcm11226696 36431172 PMC9694218

[42] Li N, Zhou H, Wu H, Wu Q, Duan M, Deng W, Tang Q. STING-IRF3 contributes to lipopolysaccharide-induced cardiac dysfunction, inflammation, apoptosis and pyroptosis by activating NLRP3. Redox Biol. 2019; 24:101215. 10.1016/j.redox.2019.101215 31121492 PMC6529775

[43] Cohen JJ, Duke RC, Fadok VA, Sellins KS. Apoptosis and programmed cell death in immunity. Annu Rev Immunol. 1992; 10:267–93. 10.1146/annurev.iy.10.040192.001411 1590988

[44] Ayala A, Perl M, Venet F, Lomas-Neira J, Swan R, Chung CS. Apoptosis in sepsis: mechanisms, clinical impact and potential therapeutic targets. Curr Pharm Des. 2008; 14:1853–9. 10.2174/138161208784980617 18691096

[45] Zechendorf E, O’Riordan CE, Stiehler L, Wischmeyer N, Chiazza F, Collotta D, Denecke B, Ernst S, Müller-Newen G, Coldewey SM, Wissuwa B, Collino M, Simon TP, et al. Ribonuclease 1 attenuates septic cardiomyopathy and cardiac apoptosis in a murine model of polymicrobial sepsis. JCI Insight. 2020; 5:e131571. 10.1172/jci.insight.131571 32213712 PMC7205433

[46] Jiang L, Zhang L, Yang J, Shi H, Zhu H, Zhai M, Lu L, Wang X, Li XY, Yu S, Liu J, Duan W. 1-Deoxynojirimycin attenuates septic cardiomyopathy by regulating oxidative stress, apoptosis, and inflammation via the JAK2/STAT6 signaling pathway. Biomed Pharmacother. 2022; 155:113648. 10.1016/j.biopha.2022.113648 36108388

[47] Weber SU, Schewe JC, Lehmann LE, Müller S, Book M, Klaschik S, Hoeft A, Stüber F. Induction of Bim and Bid gene expression during accelerated apoptosis in severe sepsis. Crit Care. 2008; 12:R128. 10.1186/cc7088 18925930 PMC2592767

[48] Han X, Chen D, Liufu N, Ji F, Zeng Q, Yao W, Cao M. MG53 Protects against Sepsis-Induced Myocardial Dysfunction by Upregulating Peroxisome Proliferator-Activated Receptor-α. Oxid Med Cell Longev. 2020; 2020:7413693. 10.1155/2020/7413693 32908637 PMC7474382

[49] Li X, Luo J, Li Y, Jia L, Li Y, Ye S, Liu L, Yu Y, Lu Y, Luan Y. Macrophage-Derived Exosomes in TLR9-/- Mice Ameliorate Sepsis-Induced Mitochondrial Oxidative Stress and Apoptosis in Cardiomyocytes. Oxid Med Cell Longev. 2022; 2022:5719974. 10.1155/2022/5719974 36225174 PMC9550441

[50] Bi CF, Liu J, Yang LS, Zhang JF. Research Progress on the Mechanism of Sepsis Induced Myocardial Injury. J Inflamm Res. 2022; 15:4275–90. 10.2147/JIR.S374117 35923903 PMC9342248

[51] Choi AM, Ryter SW, Levine B. Autophagy in human health and disease. N Engl J Med. 2013; 368:651–62. 10.1056/NEJMra1205406 23406030

[52] Liang XH, Kleeman LK, Jiang HH, Gordon G, Goldman JE, Berry G, Herman B, Levine B. Protection against fatal Sindbis virus encephalitis by beclin, a novel Bcl-2-interacting protein. J Virol. 1998; 72:8586–96. 10.1128/JVI.72.11.8586-8596.1998 9765397 PMC110269

[53] Kabeya Y, Mizushima N, Yamamoto A, Oshitani-Okamoto S, Ohsumi Y, Yoshimori T. LC3, GABARAP and GATE16 localize to autophagosomal membrane depending on form-II formation. J Cell Sci. 2004; 117:2805–12. 10.1242/jcs.01131 15169837

[54] Kabeya Y, Mizushima N, Ueno T, Yamamoto A, Kirisako T, Noda T, Kominami E, Ohsumi Y, Yoshimori T. LC3, a mammalian homologue of yeast Apg8p, is localized in autophagosome membranes after processing. EMBO J. 2000; 19:5720–8. 10.1093/emboj/19.21.5720 11060023 PMC305793

[55] Turco E, Savova A, Gere F, Ferrari L, Romanov J, Schuschnig M, Martens S. Reconstitution defines the roles of p62, NBR1 and TAX1BP1 in ubiquitin condensate formation and autophagy initiation. Nat Commun. 2021; 12:5212. 10.1038/s41467-021-25572-w 34471133 PMC8410870

[56] Chien WS, Chen YH, Chiang PC, Hsiao HW, Chuang SM, Lue SI, Hsu C. Suppression of autophagy in rat liver at late stage of polymicrobial sepsis. Shock. 2011; 35:506–11. 10.1097/SHK.0b013e31820b2f05 21263383

[57] Takahashi W, Watanabe E, Fujimura L, Watanabe-Takano H, Yoshidome H, Swanson PE, Tokuhisa T, Oda S, Hatano M. Kinetics and protective role of autophagy in a mouse cecal ligation and puncture-induced sepsis. Crit Care. 2013; 17:R160. 10.1186/cc12839 23883625 PMC4056358

[58] Tian W, Liu SY, Zhang M, Meng JR, Tang N, Feng YD, Sun Y, Gao YY, Zhou L, Cao W, Li XQ. TRPC1 contributes to endotoxemia-induced myocardial dysfunction via mediating myocardial apoptosis and autophagy. Pharmacol Res. 2022; 181:106262. 10.1016/j.phrs.2022.106262 35598715

[59] Pang J, Peng H, Wang S, Xu X, Xu F, Wang Q, Chen Y, Barton LA, Chen Y, Zhang Y, Ren J. Mitochondrial ALDH2 protects against lipopolysaccharide-induced myocardial contractile dysfunction by suppression of ER stress and autophagy. Biochim Biophys Acta Mol Basis Dis. 2019; 1865:1627–41. 10.1016/j.bbadis.2019.03.015 30946956

[60] Yang J, Pi C, Wang G. Inhibition of PI3K/Akt/mTOR pathway by apigenin induces apoptosis and autophagy in hepatocellular carcinoma cells. Biomed Pharmacother. 2018; 103:699–707. 10.1016/j.biopha.2018.04.072 29680738

[61] Zheng X, Li W, Xu H, Liu J, Ren L, Yang Y, Li S, Wang J, Ji T, Du G. Sinomenine ester derivative inhibits glioblastoma by inducing mitochondria-dependent apoptosis and autophagy by PI3K/AKT/mTOR and AMPK/mTOR pathway. Acta Pharm Sin B. 2021; 11:3465–80. 10.1016/j.apsb.2021.05.027 34900530 PMC8642618

[62] Li T, Qian Y, Miao Z, Zheng P, Shi T, Jiang X, Pan L, Qian F, Yang G, An H, Zheng Y. Xuebijing Injection Alleviates Pam3CSK4-Induced Inflammatory Response and Protects Mice From Sepsis Caused by Methicillin-Resistant Staphylococcus aureus. Front Pharmacol. 2020; 11:104. 10.3389/fphar.2020.00104 32153410 PMC7047170

[63] Shang X, Lin K, Yu R, Zhu P, Zhang Y, Wang L, Xu J, Chen K. Resveratrol Protects the Myocardium in Sepsis by Activating the Phosphatidylinositol 3-Kinases (PI3K)/AKT/Mammalian Target of Rapamycin (mTOR) Pathway and Inhibiting the Nuclear Factor-κB (NF-κB) Signaling Pathway. Med Sci Monit. 2019; 25:9290–8. 10.12659/MSM.918369 31806860 PMC6911307

[64] Qi Z, Wang R, Liao R, Xue S, Wang Y. Neferine Ameliorates Sepsis-Induced Myocardial Dysfunction Through Anti-Apoptotic and Antioxidative Effects by Regulating the PI3K/AKT/mTOR Signaling Pathway. Front Pharmacol. 2021; 12:706251. 10.3389/fphar.2021.706251 34366860 PMC8344844

[65] Rittirsch D, Huber-Lang MS, Flierl MA, Ward PA. Immunodesign of experimental sepsis by cecal ligation and puncture. Nat Protoc. 2009; 4:31–6. 10.1038/nprot.2008.214 19131954 PMC2754226

